# Socioeconomic status-based survival disparities and nomogram prediction for patients with multiple myeloma: Results from American and Chinese populations

**DOI:** 10.3389/fonc.2022.941714

**Published:** 2022-08-26

**Authors:** Jiaxuan Xu, Peipei Xu, Qiaoyan Han, Jingjing Sun, Bing Chen, Xiaoqing Dong

**Affiliations:** ^1^ Department of Hematology, the Affiliated Drum Tower Hospital of Nanjing University Medical School, Nanjing, China; ^2^ Department of Hematology, Jingjiang People’s Hospital, Jingjiang, China

**Keywords:** SES, multiple myeloma, nomogram, risk stratification, myeloma-specific survival

## Abstract

**Objective:**

This study aimed to comprehensively investigate the relationship between the survival differences and socioeconomic status (SES) in patients with multiple myeloma (MM) and construct a predictive nomogram to assess clinical outcomes of MM patients.

**Methods:**

The Surveillance, Epidemiology, and End Results (SEER) census tract-level SES database provides two specialized attributes: SES index and rurality. Using this database, 37,819 patients diagnosed with MM between January 2007 and December 2016 were enrolled. We evaluated the effects of SES index on overall survival (OS) and myeloma-specific survival (MSS) using Kaplan-Meier curves and Cox regression analyses. Thereafter, we included 126 patients with MM from two independent medical centers in China and divided them into training (Center 1) and validation (Center 2) cohorts. Univariate and multivariate Cox analyses were used in the training cohort to construct a nomogram for predicting clinical outcomes. Nomogram performance was assessed using the area under the curve (AUC) and calibration curves.

**Results:**

In the SEER cohort, lower SES was significantly associated with worse OS rates and MSS rates (both *P* < 0.001). Multivariate analysis confirmed SES as an independent predictor of survival. Subgroup analysis indicated an increasing linear trend in survival benefits in non-Hispanic White, married, insured, and urban populations with increasing SES (all *P* < 0.001). In the training cohort, albumin, creatinine, rurality, and SES were confirmed as independent prognostic indicators. A nomogram for OS prediction was developed using these four factors, and it showed satisfactory discrimination and calibration. The 18- and 36-month AUC values of the nomogram were 0.79 and 0.82, respectively. Based on the total nomogram points, patients were categorized into two risk levels with good separation.

**Conclusion:**

SES strongly influences survival disparities in patients with MM. Our nomogram consisting of clinical and sociodemographic characteristics can potentially predict survival outcomes.

## Introduction

Multiple myeloma (MM) is an incurable plasma cell dyscrasia that is characterized by the proliferation of clonal plasma cells, and it is the second most common hematologic malignancy (1, 2). The incidence of MM is notably high in developed and high-income countries such as Australia, the United States, and those in Western Europe (3, 4). The survival rates of patients with MM have continuously increased since 2000, with a 5-year relative survival rate of 55.6% between 2011 and 2017 (5). Improved survival in patients with MM is mainly attributed to the availability of novel therapies, including stem cell transplantation (SCT), advanced immune-modifying drugs, and proteasome inhibitors, but these are accompanied by increased treatment costs (6). Currently, survival outcomes vary substantially between individuals, which may largely depend on the recognized MM prognostic factors: age, sex, comorbidities, cytogenetics, the International Staging System (ISS) stage, response to chemotherapy, and social determinants (7).

Disparities in race, income, marital status, and insurance coverage are associated with survival in MM (8–10). Socioeconomic status (SES) and rurality are also imperative sociodemographic factors that potentially affect prognosis. A report showed that lower SES is independently associated with worse overall survival (OS) in patients with MM, when SES is estimated by household income alone (11). In the era of precision therapy, real-world data show that the impact of low SES on OS is more discernable in elderly patients (12). Additionally, survival in MM patients improved with a widening SES-level poverty gap over the last three decades (13). Nevertheless, these studies evaluated OS rather than myeloma-specific survival (MSS), which is more specific for predicting MM outcomes with less interference from other causes. In particular, the measures of SES varied considerably in previous studies, with lack of unified, professional, and standardized approaches for taking measurements. Given these limitations, we aimed to employ the Surveillance, Epidemiology, and End Results (SEER) census tract-level SES database for further demonstration.

The SEER census tract-level SES database is specifically designed to allow for improved investigation of the effects of SES on cancer survival. The census tract-level SES index is a composite of seven variables measuring different SES aspects, including median household income, median house value, median rent, percentage below 150% of the poverty line, working class percentage, unemployed percentage, and education level. These data are more reliable than the isolated methods of measuring SES (14, 15). In addition, the database provides another census tract-level attribute: rurality as measured by rural-urban commuting area codes. Researchers have examined the urban/rural differences in the survival of lung and breast cancer (16). Since few studies have focused on the impact of rurality on myeloma, to understand the relationship between rurality levels and prognosis of patients with MM, we hypothesized that rurality would serve as a prognostic factor for clinical outcomes.

Therefore, this study investigated the prognostic effects of SES and rurality on the survival of MM patients using the census tract-level SES database. Further, we developed and validated a novel nomogram using the data from patients at two independent medical centers in China. This nomogram will provide quick assessment of risk levels and individualized prediction of clinical outcomes.

## Materials and methods

### Patients and variables in the SEER cohort

In the SEER-based analysis, patient data were obtained from the specialized Census Tract-level SES and Rurality Database covering 18 cancer registry areas (excluding Alaska) using SEER*Stat software (version 8.3.9). Census tracts were categorized into SES quintiles with equal populations in each quintile within the overall area or in each registry. For instance, the first quintile (Q1, the group with the lowest SES) refers to the 20th percentile or lower, and the fifth quintile (Q5, the group with the highest SES) refers to the 80th percentile or higher. MM cases were identified using the International Classification of Disease for Oncology, Third Edition (ICD-O-3) histologic code 9732, and primary site code C42.1. We initially screened 42,210 patients diagnosed with MM between January 2007 and December 2016 according to the following inclusion criteria: (a) non-autopsy/death certificate-only cases, (b) unambiguous insurance information, and (c) first primary tumor. Subsequently, 37,819 patients were enrolled in the cohort for further research, grouped with SES quintiles by the baseline characteristics, after excluding the following cases: (a) unknown race (n=236); (b) non-Hispanic American Indian/Alaska Native (n=208); (c) unknown marital status (n=2,027); and (d) missing or no match for SES quintile (n=1,920).

We extracted the following sociodemographic variables from the cohort: age at diagnosis, year of diagnosis, sex, race, insurance status, marital status, rurality, and SES index. Race included four categories: non-Hispanic white (NHW), non-Hispanic black (NHB), non-Hispanic Asian or Pacific Islander (NHAPI), and Hispanic. Insurance status was categorized as insured, uninsured, or Medicaid. Marital status was defined as married (including separated), divorced, single (including unmarried or domestic partner), or widowed. According to the rural-urban commuting area codes, rurality was classified as rural or urban. Regarding the survival variables, OS was defined as the interval between diagnosis and death from any cause. MSS was defined as the interval between diagnosis and death due to myeloma.

### Patients and variables in the real-world Chinese cohorts

We retrospectively included 126 patients with newly diagnosed MM from two cancer centers in China (Jingjiang People’s Hospital, from January 2012 to November 2021; Nanjing Drum Tower Hospital, from May 2016 to June 2019). All patients were diagnosed according to the current International Myeloma Working Group consensus recommendations (17). We collected and analyzed the following patient-specific information: age, sex, bone marrow plasma cells (BMPC), albumin (ALB), β2-microglobulin (β2-MG), hemoglobin (HGB), creatinine (CREAT), history of hypertension, diabetes, smoking, insurance status, employment status, rurality, and SES. Here, SES was evaluated based on each patient’s occupation, place of residence, and the ability to pay for treatment. Patients were divided equally into two groups.

### Nomogram development and validation

We divided the enrolled patients into training and validation sets according to the medical centers. Patients registered at Jingjiang People’s Hospital (Center 1) were selected as the training cohort (n=85), and patients registered at Nanjing Drum Tower Hospital (Center 2) were selected as the validation cohort (n=41). Variables with statistical significance in the multivariate analysis were used to create the nomogram of the training cohort. The receiver operating characteristic (ROC) curves with the area under the curve (AUC) values were employed in both the training and validation cohorts for validity and sensitivity. To measure accuracy, we constructed calibration plots with 1,000 bootstrap resamples to observe errors between the actual and predicted survival rates. Moreover, the stratification of risk levels was constructed based on the nomogram total scores.

### Statistical analysis

Baseline characteristics of patients were presented as a proportion for categorical variables. The chi-squared test was used to compare the distribution of patient characteristics between the training and validation cohorts. Survival analysis was conducted using the Kaplan-Meier method and assessed using a log-rank test. Univariate and multivariate Cox proportional-hazards models were applied to evaluate the hazard ratio (HR) with corresponding 95% confidence interval (CI). The above analyses were performed using GraphPad Prism 8 and R software (Version 4.0.2). Results were considered statistically significant when the two-tailed *P-*value was less than 0.05.

## Results

### Sociodemographic characteristics of SEER patients

Baseline characteristics of the 37,819 patients in the SEER cohort are summarized in **Table 1**. The patients were divided into five groups according to SES quintiles: 7,365 in quintile 1 (Q1, lowest), 7,236 in quintile 2 (Q2, lower), 7,382 in quintile 3 (Q3, medium), 7,805 in quintile 4 (Q4, higher), and 8,031 in quintile 5 (Q5, highest). Patients in high SES groups (Q4 and Q5) were more likely to be male, NHW, insured, and married, and tended to reside in urban tracts. In the lowest SES group (Q1), the relative proportions of those designated as NHB, Medicaid, or single were the largest.

**Table 1:** Baseline sociodemographic characteristics of MM patients in the SEER cohort, grouped by SES index.

Variables	Overall	Q1 (lowest)	Q2 (lower)	Q3 (medium)	Q4 (higher)	Q5 (highest)
	N=37819 (%)	N=7365 (%)	N=7236 (%)	N=7382 (%)	N=7805 (%)	N=8031 (%)
Age (years)						
<60	10417 (27.5%)	2131 (28.9%)	2006 (27.7%)	2001 (27.1%)	2051 (26.3%)	2228 (27.7%)
60-69	11150 (29.5%)	2188 (29.7%)	2046 (28.3%)	2172 (29.4%)	2372 (30.4%)	2372 (29.5%)
70-79	9852 (26.1%)	1949 (26.5%)	1924 (26.6%)	1916 (26.0%)	2037 (26.1%)	2026 (25.2%)
≥80	6400 (16.9%)	1097 (14.9%)	1260 (17.4%)	1293 (17.5%)	1345 (17.2%)	1405 (17.5%)
Year of diagnosis						
2007-2011	17349 (45.9%)	3332 (45.2%)	3352 (46.3%)	3307 (44.8%)	3618 (46.4%)	3740 (46.6%)
2012-2016	20470 (54.1%)	4033 (54.8%)	3884 (53.7%)	4075 (55.2%)	4187 (53.6%)	4291 (53.4%)
Sex						
Male	20762 (54.9%)	3806 (51.7%)	3883 (53.7%)	4114 (55.7%)	4401 (56.4%)	4558 (56.8%)
Female	17057 (45.1%)	3559 (48.3%)	3353 (46.3%)	3268 (44.3%)	3404 (43.6%)	3473 (43.2%)
Race						
NHW	23116 (61.1%)	2581 (35.0%)	4135 (57.1%)	4735 (64.1%)	5518 (70.7%)	6147 (76.5%)
NHB	7893 (20.9%)	3347 (45.4%)	1709 (23.6%)	1320 (17.9%)	909 (11.6%)	608 (7.6%)
NHAPI	2285 (6.0%)	206 (2.8%)	301 (4.2%)	458 (6.2%)	587 (7.5%)	733 (9.1%)
Hispanic	4525 (12.0%)	1231 (16.7%)	1091 (15.1%)	869 (11.8%)	791 (10.1%)	543 (6.8%)
Insurance status						
Insured	32166 (85.1%)	5352 (72.7%)	5914 (81.7%)	6406 (86.8%)	7014 (89.9%)	7480 (93.1%)
Uninsured	996 (2.6%)	341 (4.6%)	231 (3.2%)	167 (2.3%)	138 (1.8%)	119 (1.5%)
Medicaid	4657 (12.3%)	1672 (22.7%)	1091 (15.1%)	809 (11.0%)	653 (8.4%)	432 (5.4%)
Marital status						
Married	23144 (61.2%)	3615 (49.1%)	4179 (57.8%)	4599 (62.3%)	5094 (65.3%)	5657 (70.4%)
Divorced	3577 (9.5%)	860 (11.7%)	746 (10.3%)	704 (9.5%)	708 (9.1%)	559 (7.0%)
Single	5639 (14.9%)	1636 (22.2%)	1173 (16.2%)	1013 (13.7%)	953 (12.2%)	864 (10.8%)
Widowed	5459 (14.4%)	1254 (17.0%)	1138 (15.7%)	1066 (14.4%)	1050 (13.5%)	951 (11.8%)
Rurality						
Urban	34978 (92.5%)	6291 (85.4%)	6234 (86.2%)	6792 (92.0%)	7655 (98.1%)	8006 (99.7%)
Rural	2841 (7.5%)	1074 (14.6%)	1002 (13.8%)	590 (8.0%)	150 (1.9%)	25 (0.3%)

Q1, quintile 1; Q2, quintile 2; Q3, quintile 3; Q4, quintile 4; Q5, quintile 5; NHW, non-Hispanic White; NHB, non-Hispanic Black; NHAPI, Non-Hispanic Asian or Pacific Islander.

### Survival analysis of the SEER cohort

**Figure 1:**
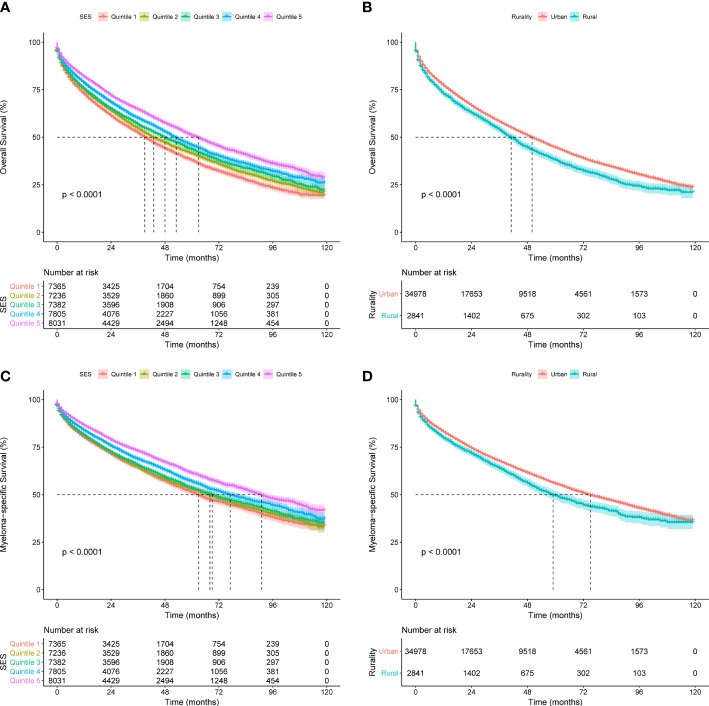
Kaplan-Meier survival curves of the SEER cohort for **(A)** OS stratified by SES quintiles, **(B)** OS stratified by rurality, **(C)** MSS stratified by SES quintiles, and **(D)** MSS stratified by rurality.

To assess the effects of SES and rurality on OS and MSS, Kaplan-Meier survival curves stratified by SES quintiles and rurality were analyzed. Patients in the highest SES group (Q5) had a median OS of 63 months, which was much higher than that of the other four groups (53, 48, 43, and 39 months, for Q4 to Q1, respectively, *P* < 0.001) (**Figure 1A**). Patients in urban tracts exhibited higher median OS than those in rural tracts (50 vs. 41 months, *P* < 0.001) (**Figure 1B**). Similarly, patients with highest SES had a median MSS of 91 months, which was quite higher than that of the other four groups (77, 69, 68, and 63 months, for Q4 to Q1, respectively, *P* < 0.001) (**Figure 1C**). Patients in urban tracts had a higher median MSS time than those in rural tracts (75 vs. 59 months, *P* < 0.001) (**Figure 1D**).

**Figure 2:**
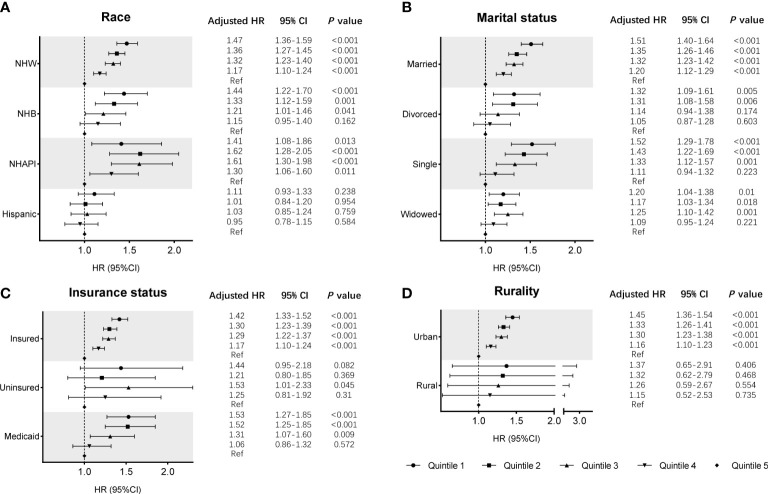
Subgroup analyses of the effects of SES on MSS in the SEER cohort, according to the subgroups of **(A)** race, **(B)** marital status, **(C)** insurance status, and **(D)** rurality, using multivariate Cox regression analysis with adjusted covariables.

Cox regression analysis identified the prognostic values of these sociodemographic factors for OS (**Table 2**) and MSS (**Table 3**). All variables except sex proved to be significantly associated with survival outcomes in the univariate analysis. Furthermore, age, year of diagnosis, sex, race, insurance status, marital status, and SES index were independent prognostic indicators of both OS and MSS in the multivariate Cox proportional-hazards models. Notably, compared with the Q1 group, the risk of a poor MSS gradually decreased from Q2 to Q5 (adjusted HR, Q2: 0.93, *P* = 0.006; Q3: 0.90, *P* < 0.001; Q4: 0.81 P < 0.001; Q5: 0.69, *P* < 0.001). To further visualize the effect of SES on MSS in subgroups, forest plots displayed the HRs by SES quintiles within the race, marital status, insurance status, and rurality groups (**Figures 2A–D**). In the NHW, married, insured, and urban groups, SES had the most significant effect on prognosis (all *P* < 0.001).

**Table 2:** Univariate and multivariate Cox regression analysis for OS in the SEER cohort.

Characteristics	Levels	Univariate analysis			Multivariate analysis		
		HR	95% CI	*P* value	Adjusted HR	95% CI	*P* value
Age	<60 years	Ref			Ref		
	60-69 years	1.39	1.33-1.45	<0.001	1.44	1.38-1.51	<0.001
	70-79 years	2.22	2.12-2.31	<0.001	2.30	2.20-2.40	<0.001
	≥80 years	4.13	3.95-4.32	<0.001	4.18	3.99-4.39	<0.001
Year of diagnosis	2007-2011	Ref			Ref		
	2012-2016	0.87	0.84-0.90	<0.001	0.87	0.84-0.90	<0.001
Sex	Male	Ref			Ref		
	Female	0.96	0.93-0.99	0.003	0.83	0.81-0.86	<0.001
Race	NHW	Ref			Ref		
	NHB	0.92	0.88-0.95	<0.001	0.88	0.85-0.92	<0.001
	NHAPI	0.90	0.84-0.95	<0.001	0.91	0.85-0.97	0.006
	Hispanic	0.95	0.91-0.99	0.027	0.93	0.88-0.98	0.003
Insurance status	Insured	Ref			Ref		
	Uninsured	0.87	0.79-0.95	0.003	1.18	1.07-1.30	0.001
	Medicaid	1.30	1.25-1.36	<0.001	1.37	1.31-1.43	<0.001
Marital status	Married	Ref			Ref		
	Divorced	1.16	1.10-1.22	<0.001	1.23	1.17-1.29	<0.001
	Single	1.16	1.11-1.21	<0.001	1.28	1.23-1.34	<0.001
	Widowed	1.95	1.88-2.03	<0.001	1.26	1.21-1.32	<0.001
Rurality	Urban	Ref			Ref		
	Rural	1.18	1.12-1.24	<0.001	0.99	0.94-1.05	0.767
SES	Quintile 1	Ref			Ref		
	Quintile 2	0.91	0.87-0.95	<0.001	0.89	0.85-0.94	<0.001
	Quintile 3	0.86	0.82-0.90	<0.001	0.85	0.81-0.89	<0.001
	Quintile 4	0.78	0.75-0.82	<0.001	0.78	0.74-0.82	<0.001
	Quintile 5	0.68	0.65-0.71	<0.001	0.68	0.64-0.71	<0.001

OS, overall survival; NHW, non-Hispanic White; NHB, non-Hispanic Black; NHAPI, Non-Hispanic Asian or Pacific Islander.

**Table 3:** Univariate and multivariate Cox regression analysis for MSS in the SEER cohort.

Characteristics	Levels	Univariate analysis			Multivariate analysis		
		HR	95% CI	*P* value	Adjusted HR	95% CI	*P* value
Age	<60 years	Ref			Ref		
	60-69 years	1.31	1.25-1.38	<0.001	1.35	1.28-1.42	<0.001
	70-79 years	1.99	1.89-2.09	<0.001	2.03	1.93-2.14	<0.001
	≥80 years	3.52	3.35-3.71	<0.001	3.52	3.32-3.72	<0.001
Year of diagnosis	2007-2011	Ref			Ref		
	2012-2016	0.86	0.82-0.89	<0.001	0.85	0.82-0.89	<0.001
Sex	Male	Ref			Ref		
	Female	1.00	0.97-1.04	0.886	0.9	0.87-0.93	<0.001
Race	NHW	Ref			Ref		
	NHB	0.86	0.82-0.90	<0.001	0.82	0.79-0.87	<0.001
	NHAPI	0.90	0.84-0.97	0.008	0.92	0.85-0.99	0.026
	Hispanic	0.95	0.90-1.00	0.053	0.92	0.87-0.98	0.006
Insurance status	Insured	Ref			Ref		
	Uninsured	0.91	0.81-1.01	0.088	1.20	1.07-1.34	0.001
	Medicaid	1.27	1.20-1.33	<0.001	1.34	1.27-1.41	<0.001
Marital status	Married	Ref			Ref		
	Divorced	1.13	1.07-1.20	<0.001	1.19	1.12-1.26	<0.001
	Single	1.12	1.06-1.17	<0.001	1.22	1.16-1.29	<0.001
	Widowed	1.82	1.74-1.91	<0.001	1.21	1.15-1.27	<0.001
Rurality	Urban	Ref			Ref		
	Rural	1.17	1.10-1.25	<0.001	0.99	0.93-1.06	0.741
SES	Quintile 1	Ref			Ref		
	Quintile 2	0.96	0.91-1.01	0.136	0.93	0.88-0.98	0.006
	Quintile 3	0.93	0.88-0.98	0.010	0.90	0.85-0.96	<0.001
	Quintile 4	0.83	0.79-0.88	<0.001	0.81	0.76-0.85	<0.001
	Quintile 5	0.72	0.68-0.76	<0.001	0.69	0.65-0.74	<0.001

MSS, myeloma-specific survival; NHW, non-Hispanic White; NHB, non-Hispanic Black; NHAPI, Non-Hispanic Asian or Pacific Islander.

### Baseline characteristics and survival of Chinese patients

The clinical and demographic characteristics of the training and validation cohorts are described in **Table 4**. A total of 126 eligible patients with MM were included in this study, with 66 (52.4%) patients aged 65 years or older and 48 (38.1%) female patients. Overall, 105 (83.3%) patients received proteasome inhibitor (PI)-based treatment regimens (bortezomib or carfilzomib), while other patients were treated with traditional medication. There were 50 (39.7%) patients with high insurance coverage, 40 (31.7%) employed patients, and 48 (38.1%) urban residents. Cases were separated into two cohorts with 85 cases from Center 1 assigned to the training cohort, and 41 patients from Center 2 assigned to the validation cohort. No significant differences were observed between the two cohorts by any of the included variables.

**Table 4:** Baseline clinical and sociodemographic characteristics of MM patients in the two-center cohorts.

Characteristics	Entire cohort	Training cohort (Center 1)	Validation cohort (Center 2)	*P* value
	N=126 (%)	N=85 (%)	N=41 (%)	
Age (years)				0.058
<65	60 (47.6%)	35 (41.2%)	25 (61.0%)	
≥65	66 (52.4%)	50 (58.8%)	16 (39.0%)	
Sex				1.000
Male	78 (61.9%)	53 (62.4%)	25 (61.0%)	
Female	48 (38.1%)	32 (37.6%)	16 (39.0%)	
M-protein subtype				0.659
IgG	52 (41.3%)	32 (37.6%)	20 (48.8%)	
IgA	41 (32.5%)	30 (35.3%)	11 (26.8%)	
FLC	27 (21.4%)	19 (22.4%)	8 (19.5%)	
Other	6 (4.76%)	4 (4.71%)	2 (4.88%)	
ISS stage				0.226
I	27 (21.4%)	17 (20.0%)	10 (24.4%)	
II	55 (43.7%)	34 (40.0%)	21 (51.2%)	
III	44 (34.9%)	34 (40.0%)	10 (24.4%)	
BMPC (%)				0.058
<25	72 (57.1%)	54 (63.5%)	18 (43.9%)	
≥25	54 (42.9%)	31 (36.5%)	23 (56.1%)	
ALB (g/dL)				0.848
<3	37 (29.4%)	24 (28.2%)	13 (31.7%)	
≥3	89 (70.6%)	61 (71.8%)	28 (68.3%)	
β2-MG (mg/L)				0.128
<5.5	82 (65.1%)	51 (60.0%)	31 (75.6%)	
≥5.5	44 (34.9%)	34 (40.0%)	10 (24.4%)	
HGB (g/dL)				0.542
<10	89 (70.6%)	62 (72.9%)	27 (65.9%)	
≥10	37 (29.4%)	23 (27.1%)	14 (34.1%)	
CREAT (mg/dL)				0.067
<2	104 (82.5%)	66 (77.6%)	38 (92.7%)	
≥2	22 (17.5%)	19 (22.4%)	3 (7.32%)	
DM				0.762
Yes	25 (19.8%)	18 (21.2%)	7 (17.1%)	
No	101 (80.2%)	67 (78.8%)	34 (82.9%)	
HTN				0.844
Yes	43 (34.1%)	30 (35.3%)	13 (31.7%)	
No	83 (65.9%)	55 (64.7%)	28 (68.3%)	
Smoking				0.050
Yes	23 (18.3%)	20 (23.5%)	3 (7.32%)	
No	103 (81.7%)	65 (76.5%)	38 (92.7%)	
Therapy regimens				0.734
PIs-based	105 (83.3%)	72 (84.7%)	33 (80.5%)	
Traditional drugs-based	21 (16.7%)	13 (15.3%)	8 (19.5%)	
Insurance status				0.929
High	50 (39.7%)	33 (38.8%)	17 (41.5%)	
Low	76 (60.3%)	52 (61.2%)	24 (58.5%)	
Employment				0.843
Employed	40 (31.7%)	26 (30.6%)	14 (34.1%)	
Unemployed	86 (68.3%)	59 (69.4%)	27 (65.9%)	
Rurality				0.259
Urban	48 (38.1%)	29 (34.1%)	19 (46.3%)	
Rural	78 (61.9%)	56 (65.9%)	22 (53.7%)	
SES				1.000
High	64 (50.8%)	43 (50.6%)	21 (51.2%)	
Low	62 (49.2%)	42 (49.4%)	20 (48.8%)	

ISS, International Staging System; BMPC, bone marrow plasma cells; ALB, albumin; β2-MG, β2-microglobulin; HGB, hemoglobin; CREAT, creatinine; DM, diabetes mellitus; HTN, hypertension; PIs, proteasome inhibitors. P value is for comparison between the training cohort and validation cohort.

**Figure 3:**
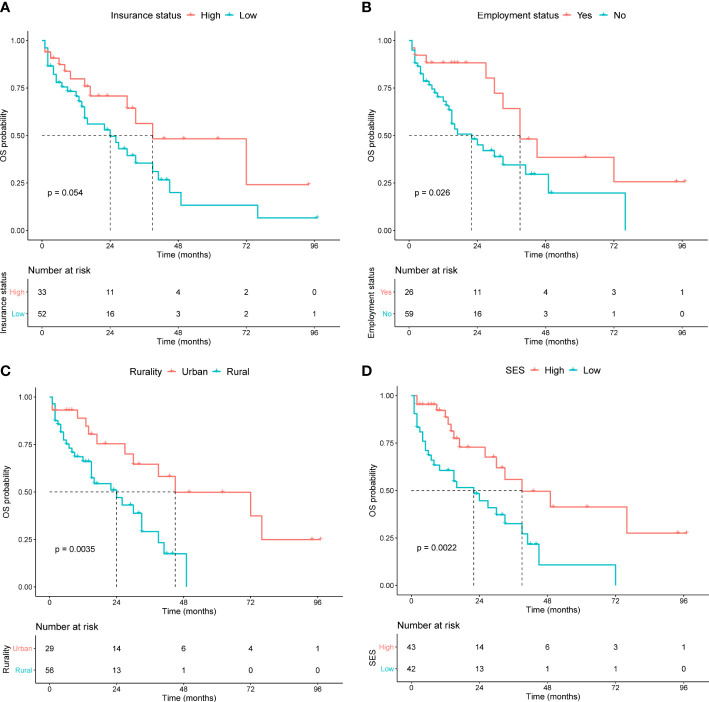
Kaplan-Meier survival curves in the training cohort for OS stratified by **(A)** insurance status, **(B)** employment status, **(C)** rurality, and **(D)** SES.

Kaplan−Meier curves were generated to evaluate the prognostic value of the socioeconomic factors. Insurance status (*P* = 0.05, **Figure 3A**), employment status (*P* = 0.03, **Figure 3B**), rurality (*P* = 0.004, **Figure 3C**), and SES (*P* = 0.002, **Figure 3D**) were linked to survival disparities in OS. Univariate and multivariate analyses were used to identify the prognostic effect of each factor in the training cohort (**Table 5**). ALB, CREAT, ISS stage, employment, rurality, and SES were correlated with OS in the univariate Cox analysis. Then, multivariate analysis confirmed that ALB, CREAT, rurality, and SES could serve as independent prognostic indictors of OS in patients with MM. ALB <3 g/dL (*P* = 0.027) or CREAT ≥2 mg/dL (*P* = 0.019) indicated worse outcomes in OS. Moreover, patients with low SES (*P* = 0.005) and those living in rural areas (*P* = 0.023) had a worse prognosis.

**Table 5:** Univariate and multivariate Cox regression analysis for OS in the training cohort.

Characteristics	Univariate analysis			Multivariate analysis		
	HR	95% CI	*P* value	Adjusted HR	95% CI	*P* value
Age (≥65 years vs. <65 years)	1.48	0.78-2.81	0.228			
Sex (female vs. male)	0.84	0.44-1.57	0.579			
BMPC (≥25% vs. <25%)	1.39	0.76-2.56	0.288			
HGB (≥10 vs. <10 g/dL)	0.76	0.37-1.57	0.461			
β2-MG (≥5.5 vs. <5.5 mg/L)	1.97	1.00-3.87	0.051			
ALB (≥3 vs. <3 g/dL)	0.49	0.26-0.93	0.028	0.39	0.17-0.90	0.027
CREAT (≥2 vs. <2 mg/dL)	2.33	1.11-4.89	0.025	3.10	1.20-7.98	0.019
ISS stage (II vs. I)	2.03	0.85-4.86	0.110	1.16	0.40-3.34	0.788
ISS stage (III vs. I)	2.94	1.13-7.66	0.027	1.53	0.51-4.53	0.447
DM (yes vs. no)	0.99	0.48-2.02	0.972			
HTN (yes vs. no)	0.74	0.38-1.45	0.379			
Smoking (yes vs. no)	1.12	0.56-2.23	0.757			
Insurance status (low vs. high)	1.91	0.98-3.74	0.059			
Employment (unemployed vs. employed)	2.22	1.08-4.59	0.031	1.56	0.67-3.63	0.299
Rurality (rural vs. urban)	2.93	1.39-6.19	0.005	2.64	1.14-6.09	0.023
SES (low vs. high)	2.73	1.40-5.31	0.003	2.72	1.35-5.46	0.005

BMPC, bone marrow plasma cells; ALB, albumin; β2-MG, β2-microglobulin; CREAT, creatinine; HGB, hemoglobin; ISS, International Staging System; DM, diabetes mellitus; HTN, hypertension.

### Construction and validation of a nomogram

**Figure 4:**
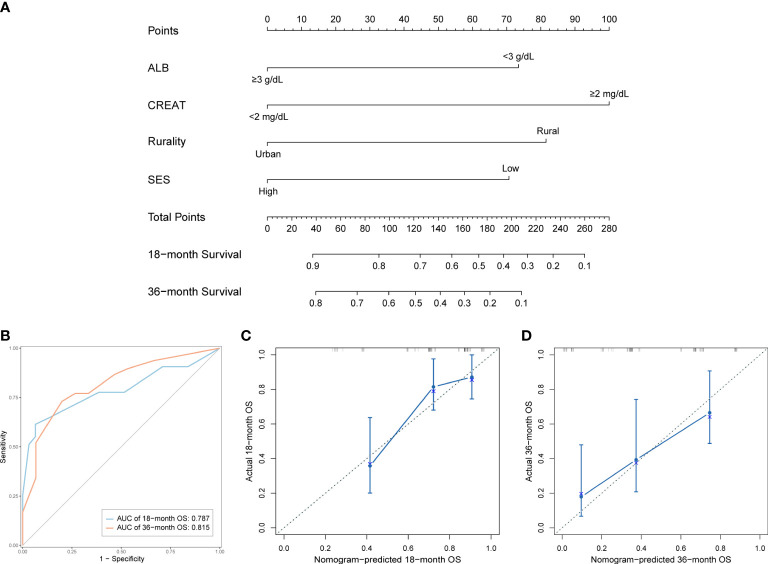
Development and validation of the prognostic nomogram in the training cohort. **(A)** Nomogram for predicting 18- and 36-month OS. **(B)** Time-dependent ROC curves and AUC values of the nomogram. **(C)** Calibration plot for predicting 18-month OS. **(D)** Calibration plot for predicting 36-month OS.

We established a predictive nomogram in the training cohort to estimate the 18- and 36-month OS probabilities (**Figure 4A**). ALB, CREAT, rurality, and SES were included in the nomogram. Different categories of these risk factors could be projected onto matching scores, which were added up to correspond to specific survival probabilities. The 18- and 36-month AUC values of the nomogram were 0.79 and 0.82, respectively, in the training cohort (**Figure 4B**) and 0.90 and 0.76, respectively, in the validation cohort (**Figure S1**), indicating adequate sensitivity and specificity. The calibration plots of both cohorts for 18- and 36-month OS showed close proximity of the predicted lines to the actual reference lines (**Figures 4C, D**; **Figure S2**), which confirmed the accuracy and reliability of our model.

**Figure 5:**
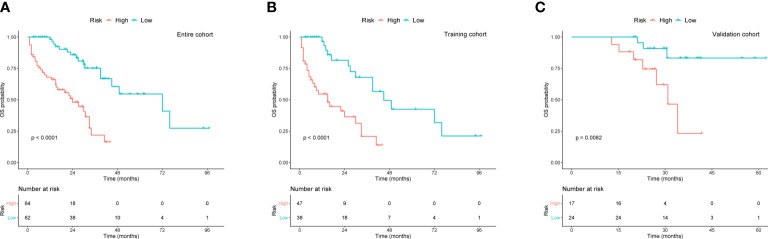
Kaplan-Meier survival curves for OS based on risk levels in **(A)** the entire cohort, **(B)** training cohort, and **(C)** validation cohort.

To better assist patients with MM in predicting their survival, we created a risk stratification based on the total points (TP) of the nomogram. Using the median risk score (TP: 153) of the nomogram model, all patients were divided into high- and low-risk groups. Kaplan-Meier curves were used to assess the discriminatory ability of the nomogram stratification. Compared to those with low-risk level, patients of the high-risk group showed a significantly worse OS in the entire cohort (*P* < 0.001, **Figure 5A**), training cohort (*P* < 0.001, **Figure 5B**), and validation cohort (*P* < 0.001, **Figure 5C**). These results revealed the effective discriminatory ability of the nomogram’s risk stratification.

## Discussion

Determining the role of socioeconomic factors in the survival of patients with MM is important (18), and we completed a large-scale retrospective cohort study to obtain more evidence regarding the role of SES in MM patient prognosis. Using the SEER census tract-level data, we found that lower SES and rural tracts were significantly associated with poorer OS and MSS. Subgroup analyses of the other demographic factors indicated that the impact of SES was more notable in the NHW, married, insured, and urban groups, with clear linear trends. In addition, this study enrolled two independent cohorts of Chinese patients with MM to confirm the effects of SES on survival. The nomogram and risk stratification showed satisfactory results for survival prediction and risk assessment.

In several studies, SES was an independent predictor of MM patient survival in multiple cohorts, which is in accordance with our results (11, 12, 19, 20). We provide clear evidence that SES inequalities are associated with survival differences among patients with MM, regardless of OS or MSS. A summary of the main papers regarding the association between SES and MM survival, is presented in **Table 6**. In contrast, the advantages of our SEER study include a notably large sample size, more classifications of the SES index, and specialized measures for SES. We also conducted a dual-center, real-world, cohort study of Chinese patients. Detailed clinical variables were included, and the importance of SES was confirmed after adjusting for covariates. With respect to rurality, rural patients experienced worse survival than patients in urban areas, which is consistent with current research related to residence. In both China and Queensland, Australia, rural patients were found to have worse survival across all age groups (19, 23).

**Table 6:** A summary of other major studies regarding the association of SES with OS in MM patients.

Authors	Countries	Sample size	Year of diagnosis	SES assessment	Results (HR, 95% CI, *P* value)
Fiala et al. (11)	US (SEER 18 registries)	45,505	2000-2009	Median household income based on the 2000 US Census data	Low-SES vs. high-SES: 1.18 (1.15-1.22), *P <*0.001; middle-SES vs. high-SES: 1.10 (1.07-1.13), *P <*0.001
Fiala et al. (11)	US	562	2000-2009	Median household income based on the American Community Survey	Low-SES vs. high-SES: 1.54 (1.13-2.09), *P* =0.006; middle-SES vs. high-SES: 1.25 (0.95-1.65), *P* =0.114
Hong et al. (21)	US	346	2003-2013	Median household income based on the 2010 US Census data	High-SES vs. low-SES: 1.08 (0.71-1.64), *P*=0.72; middle-SES vs. low-SES: 1.40 (0.93-2.10), *P* =0.11
Sun et al. (13)	US (SEER 9 registries)	12,969	2001-2010	County poverty rate	High-SES vs. low-middle-SES: 0.88 (0.84-0.92), *P <*0.001
Chan et al. (22)	New Zealand	1,864	2012-2016	The New Zealand Deprivation Index (NZDep2013) including income, home ownership, employment, qualifications, family structure, housing, access to transport, and access to communication	Low-SES vs. high-SES: 1.10 (1.04-1.16), *P*<0.05
Harwood et al. (19)	Australia	6,025	1982-2014	The Socio-Economic Indexes for Areas (SEIFA) index including income, education, employment, occupation, and housing	Low-SES vs. high-SES: 1.23 (1.07-1.40), *P*=0.004; middle-SES vs. high-SES: 1.04 (0.93-1.17), *P*=0.476
Intzes et al. (12)	Greece	223	2005-2019	The modified Kuppuswamy scale evaluated by marital status and median annual income	Low-SES vs. high-SES: 2.09 (1.36-3.20), *P <*0.001
Xu et al. (23)	China	773	2006-2019	Individual education level	High-SES vs. low-SES: 0.32 (0.19-0.56), *P*<0.001
Evans et al. (20)	US	2,543	2005-2015	Median household income, education level, and marital status	Low-SES vs. high-SES: 1.36 (1.04-1.77), *P*=0.025

Apart from SES and rurality, we also identified other covariates that could directly or indirectly affect survival and explain differences in clinical prognosis. Low SES in elderly cancer patients was linked to poor survival (24). Since MM is primarily a malignancy of the elderly, increased age at diagnosis accounts for a higher risk of MM, as older patients usually have more comorbidities, less social care, and worse response to therapies (25). Interestingly, although the proportion of NHB patients increased as the SES index decreased, NHB patients had a better prognosis than NHW patients. This finding is supported by a few clinical trials in which African Americans who underwent SCT or novel therapies had higher MSS and OS than White patients, with equal access to healthcare and treatment patterns in both groups (26–29). Race-related heterogeneity in biology and genomics may play an important role in the therapeutic effects and survival time of patients with MM. Previous research suggested that discrepancies in survival are mainly attributed to socioeconomic factors, especially SES, rather than race (8, 11, 26). Unmarried individuals, including divorced, single, and widowed, occupied a larger proportion within the lower SES groups and were proven to have worse survival outcomes. This phenomenon could be possibly explained by chronic psychological stress due to an unmarried status. Stress caused by anxiety, severe life events, and insufficient coping strategies accelerate the cellular aging process and tumor progression, which leads to increased cancer risk and mortality (30).

Despite the rapid emergence of new drugs and therapies in the field of MM, patients with different sociodemographic attributes are provided different therapies and experience different outcomes. Disparities in myeloma care are due to the limited access to health services for the more deprived patients. As a crucial part of the initial treatment, SCT was less likely to be given to patients with older age, low levels of education or income, or no medical insurance (31, 32). Further, patients with an unmarried status and lower household income had a higher burden of treatment costs, which may result in treatment interruptions (33). The accessibility and persistence of treatment modalities also depend on insurance status. The percentage of the insured population was larger in the higher SES groups. Those who were insured had more substantial survival gains in OS and MSS than those with uninsured or Medicaid status. With less insurance support, patients will have more obstacles in accessing qualified healthcare, social support, and advanced therapies (9). Moreover, patients with higher SES are more likely to live in urban tracts, where there is easier access to higher-volume facilities and better management (34).

In the context of sociology, patients with higher SES tend to obtain more social utility, leading to a lower risk of cancer mortality. The underlying mechanism is that inflammatory processes are involved in regulating the relationship between social support and cancer mortality, with patients at higher levels of social support and satisfaction having lower levels of inflammatory factors, including IL-6, TNF-α, CRP, and VEGF (35). From a psychiatric perspective, patients with low SES have a higher prevalence of depression (36). The interaction between SES and depressive symptoms is potentially mediated by interpersonal trust and reciprocity, or education level (37, 38). As a psychosocial stressor in cancers, depression promotes inflammatory reactions and oxidative stress, represses immune surveillance, and abnormally activates the hypothalamic-pituitary-adrenal axis, thus promoting tumor progression and a worse prognosis (39, 40).

Hence, we emphasized the importance of SES on survival and provided a pragmatic nomogram for clinicians and patients to better understand MM prognosis. Our nomogram contained both clinical and sociodemographic features, with good accuracy in both the training and validation cohorts. Once a diagnosis of MM is made, patients could easily predict survival prognosis according to their individual characteristics. Additionally, the risk stratification distinctly identifies two risk levels and displays marked differences in survival outcomes between the two populations. The nomogram along with the risk system may become a complementary tool in clinical practice, and more potential risk factors are expected to be identified and included in future research.

There were several limitations to our study. First, the SEER database does not include clinicopathological or molecular variables for MM, and we were therefore unable to assess disease-specific factors in the multivariate analysis. Second, the SES index provided by SEER was at the census-tract level instead of the individual level. Detailed individual information may afford patients with MM a more personalized prediction of their survival. Third, controversy persists in real-life when assigning causes of death to underlying diseases. For instance, it is not mentioned in SEER whether infections are considered related to death caused by myeloma, which may affect the accuracy of MSS.

Although SES is difficult to change in a short period of time by patients, more equitable access to healthcare resources could diminish its impact on disease processes. Medical institutions and clinicians should focus on addressing these discrepancies, providing effective interventions, and seeking optimal practices. The underlying pathogenesis of socioeconomic causes also requires further elucidation. Monitoring future trends in incidence and mortality within different socioeconomic groups is recommended. Considering the pronounced relationship between SES and patient survival, it would be meaningful to track and appraise the quality of life with SES changes during long-term treatment.

To summarize, our study identified SES as an independent predictor of survival in MM. Patients with a higher SES tend to have more favorable survival outcomes. The prognostic nomogram and risk stratification model are reliable and convenient, which improves risk assessment for each patient. More effort is needed to improve survival for patients with adverse socioeconomic factors.

## Data availability statement

The original contributions presented in the study are included in the article/**Supplementary Material**. Further inquiries can be directed to the corresponding author.

## Ethics statement

Written informed consent was obtained from the individual(s) for the publication of any potentially identifiable images or data included in this article.

## Author contributions

All authors made a significant contribution to the work reported, whether in the conception, study design, execution, acquisition of data, analysis and interpretation, or in all these areas. Additionally, all authors participated in drafting, revising, and reviewing the article; giving final approval of the version to be published; agreeing on the journal to which the article has been submitted; and remaining accountable for all aspects of the work.

## Funding

This work was supported by grants from the Jiangsu Provincial Medical Innovation Team (CXTDA2017046) and the Nanjing Medical Science and Technique Development Foundation (YKK20083).

## Acknowledgments

We appreciate the SEER program for providing access to the specialized census tract-level SES and rurality data.

## Conflict of interest

The authors declare that the research was conducted in the absence of any commercial or financial relationships that could be construed as a potential conflict of interest.

## Publisher’s note

All claims expressed in this article are solely those of the authors and do not necessarily represent those of their affiliated organizations, or those of the publisher, the editors and the reviewers. Any product that may be evaluated in this article, or claim that may be made by its manufacturer, is not guaranteed or endorsed by the publisher.

## Abbreviations

MM, multiple myeloma; SES, socioeconomic status; SEER, Surveillance, Epidemiology, and End Results; SCT, stem cell transplantation; OS, overall survival; MSS, myeloma-specific survival; HR, hazard ratio; CI, confidence interval; NHW, non-Hispanic White; NHB, non-Hispanic Black; NHAPI, non-Hispanic Asian or Pacific Islander; ROC, receiver operating characteristic; AUC, area under the curve; ISS, International Staging System; BMPC, bone marrow plasma cells; ALB, albumin; β2-MG, β2-microglobulin; HGB, hemoglobin; CREAT, creatinine; DM, diabetes mellitus; HTN, hypertension; PIs, proteasome inhibitors.
