# Supplementary material for: Socioeconomic status-based survival disparities and nomogram prediction for patients with multiple myeloma: Results from American and Chinese populations
Source: Front Oncol. 2022 Aug 26;12:941714. doi: 10.3389/fonc.2022.941714 (PMC9458969; doi:10.3389/fonc.2022.941714)

Figure S1. Time-dependent ROC curves and AUC values for evaluating the nomogram performance in the validation cohort.

Abbreviations: OS, overall survival; ROC, receiver operating characteristic; AUC, area under the curve.

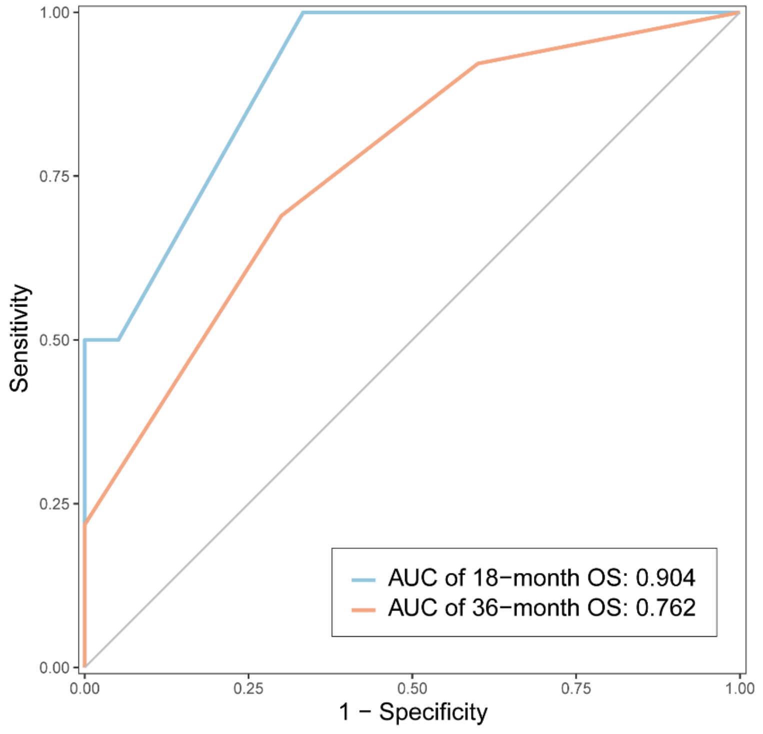

Figure S2. Calibration plots of 18- and 36-month OS in the validation cohort.

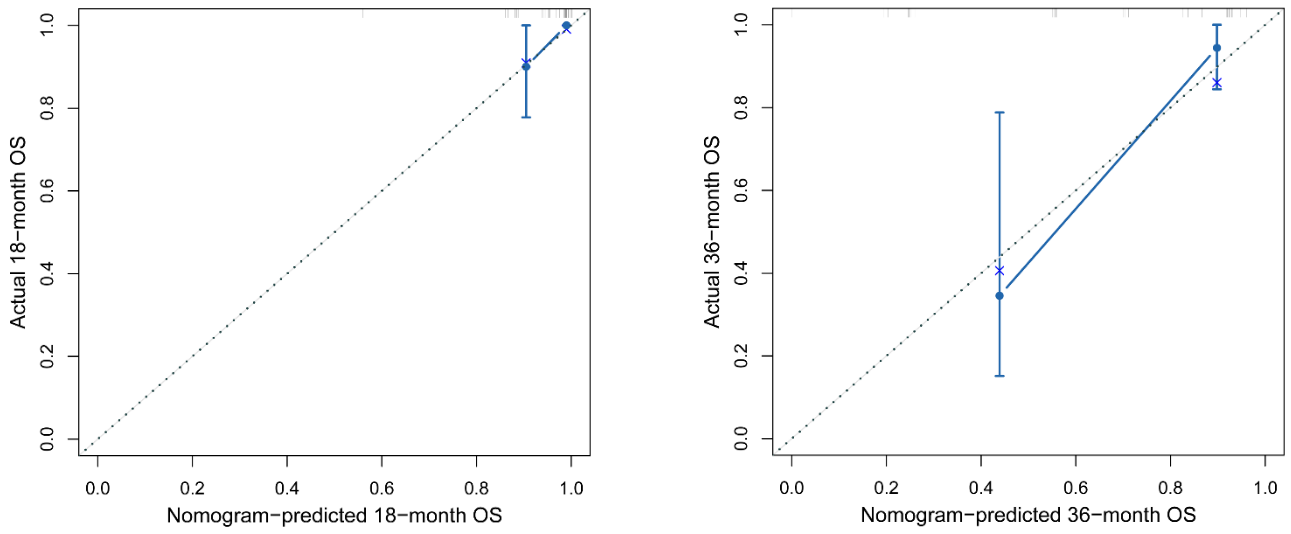

Supplement: Supplementary file 1 [file DataSheet_1.pdf]
